# Expression of terminal oxidases under nutrient-starved conditions in *Shewanella oneidensis*: detection of the A-type cytochrome *c* oxidase

**DOI:** 10.1038/srep19726

**Published:** 2016-01-27

**Authors:** Sébastien Le Laz, Arlette kpebe, Marielle Bauzan, Sabrina Lignon, Marc Rousset, Myriam Brugna

**Affiliations:** 1 CNRS, Aix-Marseille Université, Laboratoire de Bioénergétique et Ingénierie des Protéines, UMR 7281, IMM, 13402 Marseille Cedex 20, France; 2 CNRS, Aix-Marseille Université, Unité de fermentation, FR3479, IMM, 13402 Marseille Cedex 20, France; 3 CNRS, Aix-Marseille Université, Plate-forme Protéomique, FR3479, IMM, MaP IBiSA, 13402 Marseille Cedex 20, France

## Abstract

*Shewanella* species are facultative anaerobic bacteria that colonize redox-stratified habitats where O_2_ and nutrient concentrations fluctuate. The model species *Shewanella oneidensis* MR-1 possesses genes coding for three terminal oxidases that can perform O_2_ respiration: a *bd*-type quinol oxidase and cytochrome *c* oxidases of the *cbb*_3_-type and the A-type. Whereas the *bd*- and *cbb*_3_-type oxidases are routinely detected, evidence for the expression of the A-type enzyme has so far been lacking. Here, we investigated the effect of nutrient starvation on the expression of these terminal oxidases under different O_2_ tensions. Our results reveal that the *bd*-type oxidase plays a significant role under nutrient starvation in aerobic conditions. The expression of the *cbb*_3_-type oxidase is also modulated by the nutrient composition of the medium and increases especially under iron-deficiency in exponentially growing cells. Most importantly, under conditions of carbon depletion, high O_2_ and stationary-growth, we report for the first time the expression of the A-type oxidase in *S. oneidensis,* indicating that this terminal oxidase is not functionally lost. The physiological role of the A-type oxidase in energy conservation and in the adaptation of *S. oneidensis* to redox-stratified environments is discussed.

Members of the genus *Shewanella* constitute a physiologically and ecologically diverse group of facultative anaerobic bacteria which are widely distributed in aquatic and sedimentary systems^1^,^2^. A remarkable respiratory versatility allowing the use of a wide variety of electron acceptors combined with a complex chemotaxis system to locate the most favorable conditions for growth represent formidable assets for *Shewanella* species to colonize stratified environments^2^,^3^,^4^. While anaerobic respiration in *Shewanella* has received considerable attention given the biotechnological potential for the bioremediation of various environmental pollutants^1^, only a few studies have focused on aerobic respiration. Yet, in habitats that span from anaerobic to highly aerobic conditions, adaptation to differences in temporal and spatial O_2_ concentrations is clearly key to the ecological success of the *Shewanella* species and underscores the importance of understanding the mechanisms involved.

During aerobic respiration, terminal oxidases couple the oxidation of a quinol or a *c*-type cytochrome to the reduction of O_2_, producing water. In prokaryotes, terminal oxidases are grouped in two major families: the *bd*-type oxidases and the heme-copper oxidases (HCOs). Cytochrome *bd*-type oxidases use quinol as electron donor, function bioenergetically less efficiently than HCOs and are generally induced under O_2_-limited conditions and/or a wide variety of stress conditions^5^,^6^. HCOs are divided into three subfamilies: the A subfamily including mitochondrial-like terminal oxidases (*aa*_3_-type), the B subfamily comprising terminal oxidases from extremophilic prokaryotes (*ba*_3_-type oxidases) and the *cbb*_3_-type oxidases of the C subfamily^7^,^8^. Contrary to the eukaryotic (mammalian) respiratory system where electrons are transferred to a single terminal oxidase, most bacteria possess multiple terminal oxidases that differ in proton pumping efficiency and O_2_ affinity, enabling energy production under various O_2_ tensions^9^. A-type oxidases have a low affinity for O_2_ with a proton-pumping stoichiometry of 1 H^+^ /e^−^, whereas *cbb*_3_-type oxidases are generally considered to have a high affinity for O_2_ but with a lower H^+^/e^−^ ratio of 0.5^10^,^11^. Consequently, A-type oxidases are more efficient at transducing energy than *cbb*_3_-type oxidases and are the major terminal oxidases expressed under aerobic conditions. In those bacteria that possess both an A-type and a *cbb*_3_-type oxidase, the former is expressed to support growth when O_2_ is abundant while the latter provides the capacity to respire traces of O_2_ in microaerobic conditions^12^. The genome of the model species *Shewanella oneidensis* MR-1 encodes three terminal oxidases: a *bd*-type quinol oxidase and two HCOs, a *cbb*_3_-type oxidase and an A-type cytochrome *c* oxidase (Cox)^13^,^14^. In a previous study on the multiple respiratory systems of *S. oneidensis* MR-1, it was proposed that the *cbb*_3_-type and Cox are expressed under low and high O_2_ tensions, respectively, whereas the *bd*-type oxidase would be required for growth under microaerobic conditions and/or stressful conditions^15^. Subsequently it was indeed reported that, while weakly expressed in aerobic conditions, the *bd*-type oxidase conferred resistance to nitrite in *S. oneidensis*^16^. Concerning the HCOs in *S. oneidensis*, however, genetic and biochemical analysis indicated that under both microaerobic and aerobic conditions only the *cbb*_3_-type oxidase was expressed^14^,^17^. Thus, the *cbb*_3_-type oxidase was the predominant oxidase under aerobic conditions while, unexpectedly, the Cox oxidase had no physiological significance under the tested conditions. In view of these results it was proposed that in the course of evolution the Cox was functionally lost^17^. Nevertheless, this hypothesis is refuted by the facts that (i) the entire *cox* gene cluster is conserved in the *S. oneidensis* genome, and that (ii) the Cox amino acid sequence is highly conserved as compared to phylogenetically related bacteria^14^. Up to now, *Pseudomonas aeruginosa* is the only bacterium that exhibits a HCO expression pattern similar to *S. oneidensis*. Indeed, the expression level of the genes encoding an A-type oxidase is kept low under high O_2_ tensions whereas the genes encoding the *cbb*_3_-1 oxidase are highly expressed under the same conditions^18^. Interestingly, the promoter of the A-type oxidase was found to be induced under nitrogen-, iron- and carbon starvation^18^,^19^.

In this study, we surmised that Cox could also be expressed under certain specific conditions. To verify this notion, the expression patterns of the terminal oxidases in *S. oneidensis* were investigated under nutrient-starved conditions and under different dissolved O_2_ tensions. In addition to the notable modulation of the expression of the *bd*-type and *cbb*_*3*_-type oxidases, Cox was detected in cells grown to stationary phase under carbon depletion and highly aerobic conditions. The present results constitute the first report of the expression of the A-type oxidase in *S. oneidensis*. The physiological role of Cox in the energy conservation and adaptation of *S. oneidensis* to stratified environments will be evaluated in the discussion section.

## Results

### Growth parameters of *S. oneidensis* MR-1 in rich, minimum or nutrient-depleted medium

To determine the physiological significance of the different O_2_ reductases under nutrient-starved conditions, *S. oneidensis* was grown aerobically in either rich medium (LB), minimal medium (MM3), carbon-depleted medium (MM3C^−^; MM3 without sodium DL-lactate) or an iron-depleted medium (MM3I^−^; MM3 without FeCl_2_) (see Methods section for details). The wild-type strain was characterized by measuring the growth parameters (Table 1). As expected, maximum growth rate and yield were reached when the strain was grown in LB medium. Under carbon starvation, doubling time increased twofold and the cell density at the stationary phase was threefold lower as compared to minimal medium. The effect of iron depletion on growth was however less pronounced, with a twofold lower growth yield and a doubling time only slightly decreased relative to growth in minimal medium.

### Cytochrome *c* oxidase activity in solubilised membranes of *S. oneidensis*

Cytochrome *c* oxidase activity was determined in solubilised membranes of the *S. oneidensis* MR-1 wild-type strain and the SLL01 mutant strain, which lacks the *cbb*_3_ oxidase. Cells were grown under aerobic (~95 μM O_2_) or highly aerobic (~165 μM O_2_) conditions, in rich, minimal or depleted-medium. O_2_ consumption was monitored with a Clark-type electrode using *N*,*N*,*N*′,*N*′-tetramethyl-*p*-phenylenediamine (TMPD), an artificial electron donor capable of reducing cytochrome *c* (Table 2). We previously demonstrated that TMPD is not oxidized by the *bd*-type oxidase in *S. oneidensis*^14^. Accordingly, TMPD oxidase activity only corresponds to the activity of the *cbb*_3_-type and A-type cytochrome *c* oxidases.

In the wild-type strain grown on rich medium (LB), TMPD oxidase activity was constant regardless of growth phase and O_2_ tension. During growth on minimal medium (MM3), TMPD oxidase activities were slightly lower in highly aerobic conditions than in aerobic conditions. Measured values for MM3 were approximately half those of rich medium. TMPD oxidase activities were higher under conditions of nutrient deficiency than in minimal medium, with significant differences depending on the nature of the starvation. While the activity under iron starvation (MM3I^−^) was 2–3 times higher during exponential phase compared to stationary phase, the opposite pattern was observed under carbon starvation (MM3C^−^), where TMPD oxidase activity was significantly higher during stationary phase than during exponential phase. Although no distinction is made at this point between the activities of the A-type and the *cbb*_3_-type terminal oxidases, these data highlight that cytochrome *c* oxidase activity is not only influenced by O_2_ tension and growth phase but also by the availability of carbon and iron in *S. oneidensis*.

In the *cbb*_3_ deletion mutant SLL01, TMPD oxidase activity was only detected under carbon deficiency and during stationary phase, at both aerobic and highly aerobic conditions. Since TMPD oxidase activity can only arise from the A-type oxidase in SLL01, this result directly shows that Cox is not functionally lost but can be expressed under specific culture conditions. In addition, similar variations were observed when cytochrome *c* oxidase activity was measured spectrophotometrically via the oxidation of reduced horse heart cytochrome *c* (data not shown).

### Detection of *a*-type hemes by spectroscopic and HPLC analyses

**Figure 1:**
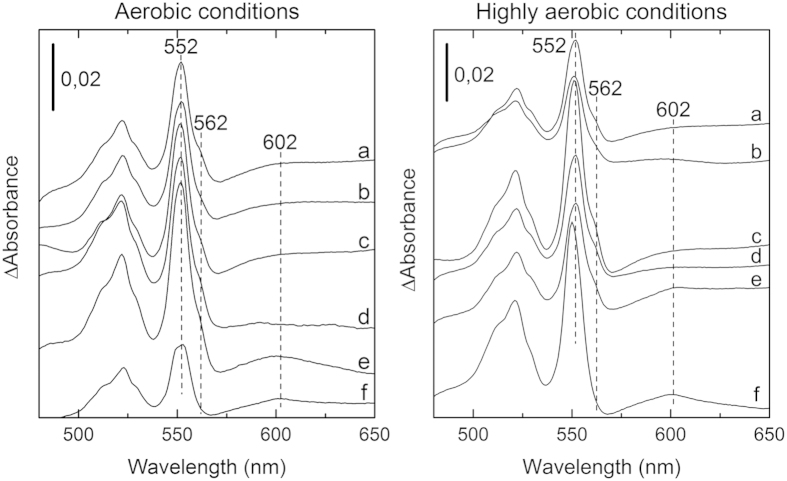
Reduced minus oxidized difference absorbance spectra of solubilised membranes from *S. oneidensis* wild-type and the *cbb*_3_ oxidase deletion strain (SLL01) grown under different O_2_ and nutrient conditions. The spectra were recorded at room temperature in the presence of 50 μM KCN. Membranes were oxidized with potassium ferricyanide and reduced with sodium ascorbate. The concentration of proteins was 6.0 mg.mL^−1^. The vertical bars indicate the absorption scale. (a) wild type in LB at stationary phase. (b) wild type in minimal medium (MM3) at stationary phase. (c) wild type in iron-depleted medium (MM3I^−^) at stationary phase. (d) wild type in carbon-depleted medium (MM3C^−^) at exponential phase. (e) wild type in carbon-depleted medium (MM3C^−^) at stationary phase. (f) SLL01 strain in carbon-depleted medium (MM3C^−^) at stationary phase.

**Figure 2:**
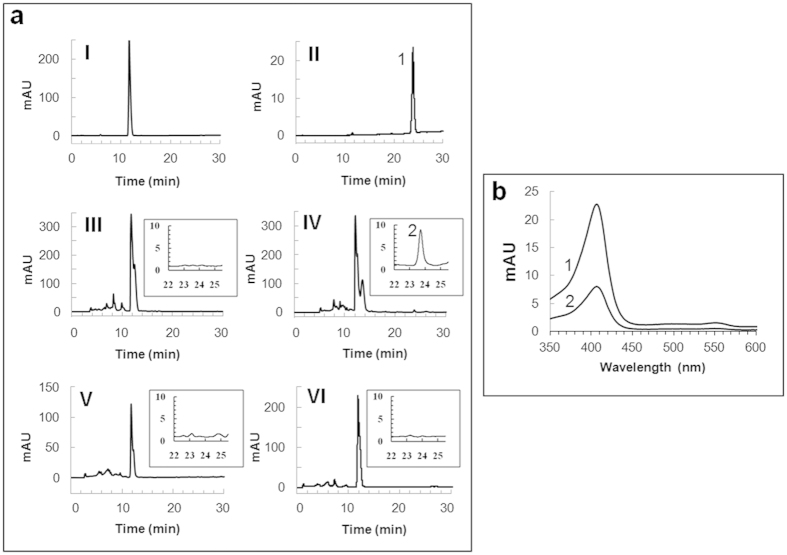
HPLC analysis of non-covalently bound hemes extracted from membranes of *S. oneidensis* grown under different O_2_ and nutrient conditions. (**a**) Heme analysis by reversed-phase HPLC and detection at 405 nm. Hemes were extracted from 10 mg of membranes of *S. oneidensis* grown under different culture conditions: highly aerobic conditions and carbon starvation at the exponential (III) and the stationary (IV) phases; highly aerobic conditions and iron starvation at the stationary phase (V); aerobic conditions and carbon starvation at the stationary phase (VI). Hemes were identified by comparison with the retention times of hemin (I) and heme *a* extracted from the *aa*_3_-type cytochrome *c* oxidase from bovine heart (II). Retention times for hemin and heme *a* were 11.7 and 23.9 min, respectively. Insets are zoom-ins of the chromatograms between 22 and 25.5 min. (**b**) Absorption spectra (photo-diode array) of peaks 1 and 2 from panels II and IV, respectively.

Since the Cox of *S. oneidensis* has so far not been characterized, the nature of its heme cofactors remained unknown. Nonetheless, the conservation of an arginine residue known to interact with the formyl group of heme *a* in *aa*_3_-type oxidases, and the presence of genes involved in heme *a* synthesis within the *cox* gene cluster suggest that subunit I of Cox contains *a*-type hemes^14^. Heme *a* is found as prosthetic group in only one class of proteins, namely the cytochrome *a*-containing respiratory oxidases in aerobic organisms^20^. Since heme *a* absorbs in the 600 nm-region, solubilised membranes from the *S. oneidensis* wild type and SLL01 *cbb*_3_-deletion mutant strains were analyzed by absorption spectroscopy in stationary phase cells (Fig. 1; also includes exponential WT cells in MM3C^−^) and also in exponentially growing cells (Fig. S1). Spectra show the presence of cytochrome *c* with peaks around 552 nm and cytochrome *b* with characteristic shoulders around 562 nm. These signals derive from several different cytochrome *c* and *b* proteins present in the membrane. In the SLL01 Δ*cbb*_3_ mutant, a spectral signal for heme *a* was detected at 602 nm in membranes from stationary-phase cells grown in carbon-depleted medium, under both aerobic and highly aerobic conditions (Fig. 1f). This observation is consistent with the TMPD oxidase activity that was measured in the same conditions and which can only arise from Cox (Table 2). In the wild-type strain, heme *a* was only clearly detected in membranes from cells grown to stationary phase under carbon starvation and highly aerobic conditions (Fig. 1 and Fig. S1). An unclearly defined putative peak at 602 nm under aerobic conditions in MM3C^−^ was observed that might be proposed to belong to heme *a* (Fig. 1e), which prompted us to further assess the presence of hemes in the membranes of *S. oneidensis* MR-1 by HPLC analysis (Fig. 2). In all culture conditions, a major elution peak was detected at around 12 min that corresponds to heme *b* as judged by the retention time (Fig. 2a) and absorption spectrum (data not shown) of hemin. An additional small peak eluting at around 24 min was identified only with hemes extracted from membranes of stationary-phase cells cultivated under carbon-depleted and highly aerobic conditions (Fig. 2a, IV). This peak was identified as heme *a*, since heme *a* extracted from the *aa*_3_-type oxidase from bovine heart exhibited an identical retention time (Fig. 2a, II) and similar spectral characteristics with an absorbance peak at 407 nm (Fig. 2b).

Overall, spectroscopic data and HPLC analysis together suggest that Cox contains heme *a* and is only expressed during the stationary phase under carbon-depleted and highly aerobic conditions in *S. oneidensis*.

### Identification of Cox by mass spectrometry

**Figure 3:**
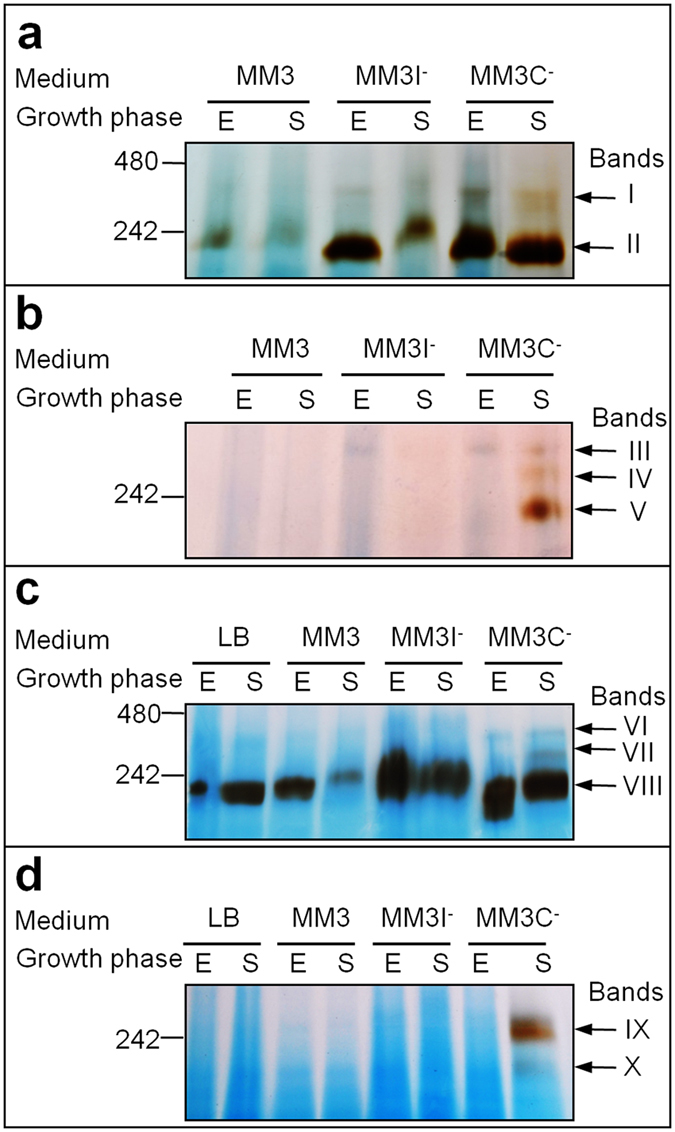
In-gel detection of cytochrome *c* oxidase activity in BN-gels run with solubilised membranes of *S. oneidensis*. Membrane proteins were prepared from *S. oneidensis* wild-type (**a,c**) and strain SLL01 lacking the *cbb*_3_-type oxidase (**b,d**) grown in aerobic (**a**,**b**) or highly aerobic (**c**,**d**) conditions. The *S. oneidensis* strains were grown on rich medium (LB), minimal medium (MM3), iron-depleted medium (MM3I^−^) or carbon-depleted medium (MM3C^−^), and cells were harvested during the exponential (E) or stationary (S) phase of growth. Total proteins (130 μg) were loaded on a 5–15% polyacrylamide gel. Bands of activity are indicated by roman numerals. Molecular mass markers are indicated in kDa.

To identify the cytochrome *c* oxidase(s) responsible for the detected TMPD oxidase activity (Table 2), membrane proteins from *S. oneidensis* strains MR-1 and SLL01 were separated via Blue Native gel electrophoresis followed by in-gel detection of cytochrome *c* oxidase activity (Fig. 3). Bands showing activity were cut out from the gel and the proteins digested by trypsin and identified by ESI-Q-ToF mass spectrometry (Table 3 for MM3C^−^ medium and Table S1 for other culture media).

Previously, we reported that the *cbb*_3_-type oxidase was the only cytochrome *c* oxidase present in the *S. oneidensis* wild-type strain grown in rich medium under aerobic conditions, and that the A-type cytochrome *c* oxidase was not present in the SLL01 strain under these conditions, despite the lack of the *cbb*_3_-type cytochrome *c* oxidase^14^. Using membranes from stationary SLL01 cells grown in rich, minimal or iron-depleted media, irrespective of the O_2_ tension, at only one molecular weight position on the gel were very faint activity bands revealed (Fig. 3b, position of band III). No cytochrome *c* oxidase peptides were identified in these bands by mass spectrometry (data not shown). At the same position, an activity band was observed in membranes from SLL01 cells growing exponentially under carbon-depleted and aerobic conditions (Fig. 3b, band III), but no cytochrome *c* oxidase was identified in this band either (Table 3). Additional bands were only observed in membranes of stationary SLL01 cells grown in carbon-depleted medium under aerobic (Fig. 3b, bands IV and V) and highly aerobic conditions (Fig. 3d, bands IX and X). Whereas in band X no cytochrome *c* oxidase was detected, bands IV, V and IX were found to contain CoxB, the subunit II of Cox (Table 3). Furthermore, CoxB was also identified in SLL01 membranes when the strain was grown to stationary phase in MM3C^−^ under microaerobic conditions (Fig. S2). In line with the presence of Cox in the mutant SLL01, TMPD oxidase activity was detected in the membranes of the bacterium in this condition (Fig. S2).

Using membranes of the wild-type strain cultivated in aerobic conditions, either one or two activity bands were revealed regardless of the culture medium and the growth phase (Fig. 3a, bands I and II). Whereas no cytochrome *c* oxidase was detected by mass spectrometry in band I, subunits II and III of the *cbb*_3_-type oxidase were identified in band II, the major activity band (Table 3 and Table S1). Nevertheless, mass spectrometry analysis of bands I and II revealed none of the Cox subunits. In-gel activity staining showed an additional band (Fig.3c, band VII) in membranes of stationary wild-type cells grown under carbon-starved and highly aerobic conditions. ESI-Q-ToF analysis indicated that these membranes contain the *cbb*_3_-type as well as the Cox terminal oxidase (Table 3). In any other condition, the *cbb*_3_-type oxidase was the only terminal oxidase identified (Table S1), not even one specific peptide belonging to Cox was found after analysis of dozens of samples. Unlike the SLL01 strain, none of the Cox subunits were detected by mass spectrometry analysis in *S. oneidensis* MR-1 cells cultivated under carbon depletion and microaerobic conditions (Fig. S2). This makes the expression of Cox at any physiologically meaningful levels in these conditions highly unlikely. As a rule, the same pattern was obtained and the same proteins were identified by mass spectrometry when TMPD was used instead of cytochrome *c* to reveal cytochrome *c* oxidase activity (data not shown).

Collectively, these results confirm that, among the tested conditions, the Cox A-type oxidase is only expressed in *S. oneidensis* under highly aerobic and carbon-depleted conditions during the stationary phase. In contrast, *S. oneidensis* membranes contain the *cbb*_3_-type oxidase whatever the culture conditions. Consequently, the *cbb*_3_-type oxidase is the only HCO present in *S. oneidensis* membranes in rich, minimal and iron-depleted media under aerobic and highly aerobic conditions.

### Binding sites of regulatory factors in the *cox* promoter

**Figure 4:**
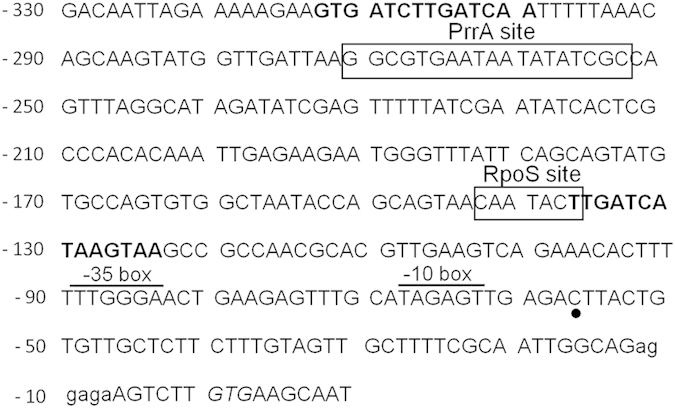
Promoter region sequence of the *cox* operon encoding the A-type cytochrome *c* oxidase. The putative Shine-Dalgarno sequence and translation start codon are in lower case and italic, respectively. Predicted Fnr-binding sites are in bold while putative RpoS-and PrrA-binding sites are boxed. Putative transcription start site, −10 and −35 boxes for the sigma 70 transcription factor are indicated by a dot and by horizontal bars respectively. Numbers indicate the position relative to the start codon.

So far, our results clearly indicate that *S. oneidensis* membranes contain Cox only during the stationary phase under carbon starvation and at high O_2_ tensions. With respect to the regulatory mechanism that enables this conditional expression, it is interesting that the promoter of the A-type oxidase in *P. aeruginosa* is induced in the same conditions^18^. In this bacterium, the *cox* promoter is activated by the stationary phase sigma factor RpoS and is repressed by the two-component regulatory system RoxSR under low O_2_ tension. Genes encoding putative RpoS (SO_3432) and PrrBA, a RoxSR analog system (SO_4173 and SO_4172) are found in the genome of *S. oneidensis* and putative binding sites for the two regulators, RpoS and PrrA, have been identified in the *cox* promoter inferred from consensus binding sequences^21^,^22^,^23^,^24^ (Fig. 4). This suggests that the regulatory mechanism involved in the expression of the A-type oxidase in *S. oneidensis* may be similar to that in *P. aeruginosa*. Nevertheless, differences between both regulatory mechanisms may exist since Cox was not detected under conditions of iron deficiency in *S. oneidensis*, while the *cox* promoter is induced in those same conditions in *P. aeruginosa*^18^. Moreover, two predicted Fnr-binding sites are present in the *cox* promoter of *S. oneidensis* whereas the *cox* genes of *P. aeruginosa* are not regulated by ANR, an analog of Fnr^18^. In addition to the putative RpoS binding site, putative −10 and −35 boxes for the sigma 70 transcription factor are present in the *cox* promoter region (Fig. 4), suggesting that both sigma factors are involved in the regulation of expression of the *cox* operon in *S. oneidensis*.

### Expression of the *bd*-type oxidase under nutrient starvation

To investigate the role of the *bd*-type oxidase in *S. oneidensis* under nutrient starvation, quinol oxidase activity was polarographically determined in solubilised membranes by measuring O_2_ reduction in the presence of ubiquinol-1 as the electron donor (Table 4). The presence of the quinol oxidase in the membranes was also analysed by ESI Q-ToF mass spectrometry (Fig. S2 and Table S2) and by light absorption spectroscopy (Fig. S3). When using minimal medium, quinol oxidase activity was significantly lower in highly aerobic conditions than in aerobic conditions. No quinol-dependent O_2_ uptake was detected in membranes from cultures under iron- and carbon starvation at high O_2_ tension. However, heme *d* was spectroscopically detected at 632 nm (Fig. S3) and CydA, one of the two subunits of the *bd*-quinol oxidase, was identified by mass spectrometry analysis in solubilised membranes of the bacterium in the same conditions (Table S2). This suggests that, for reasons that we cannot currently fathom, the membranes contain the *bd*-type oxidase but in an inactive form. Furthermore, the same situation is observed in stationary cells grown under microaerobic conditions and carbon starvation, where the *bd*-quinol oxidase was detected in the membranes but no quinol oxidase activity was measured (Fig. S2). On a more general note, the CydA subunit was identified by mass spectrometry in cytochrome *c* oxidase activity bands cut out from BN-gels (shown in Fig. 3), run with solubilised membranes of both *S. oneidensis* MR-1 and the *cbb*_3_ deletion mutant grown in all tested conditions (data not shown). This means that the *bd*-quinol oxidase is present in the membranes of *S. oneidensis* in virtually all the tested conditions.

As compared to any condition involving rich medium, quinol oxidase activity levels were much higher in aerobic cells exponentially growing in minimal medium, either iron-deplete or low carbon (Table 4). These high activities are comparable to those measured under microaerobic conditions in rich medium^14^, which leads us to believe that the *bd*-type oxidase may have an important role under aerobic nutrient starvation conditions. Nevertheless, the expression of the *bd*-type oxidase must be regulated by other factors than is the case for Cox, since quinol oxidase activity was higher under high O_2_ tensions in rich medium, and no activity was detected in cells harvested at the stationary phase under carbon starvation in aerobic conditions. It is noteworthy that the *bd* quinol oxidase activity is not at all detected under the culture conditions where Cox is expressed in *S. oneidensis* membranes.

## Discussion

In this work, we investigated the effect of nutrient starvation on the expression of the terminal oxidases in *S. oneidensis* MR-1 under different O_2_ tensions. Our results reveal that their expression is indeed not only affected by the O_2_ concentration and the growth phase but also by nutrient starvation, depending on the type of nutrient involved. The *bd* quinol oxidase was significantly induced in aerobic conditions under nutrient depletion, whereas it was only weakly expressed in rich medium^14^,^17^. This is in accordance with the fact that the enzyme is required for growth under various unfavourable conditions such as low iron availability^5^,^25^. However, the *bd* quinol oxidase was not involved in respiration under nutrient depletion in highly aerobic and microaerobic conditions. Although the *cbb*_3_-type oxidase was identified in *S. oneidensis* membranes in all tested culture conditions, its expression was still modulated by the nutrient composition of the medium, with especially iron starvation resulting in a strong expression in exponentially growing cells.

The most noteworthy outcome of this study is that the A-type cytochrome *c* oxidase in *S. oneidensis* is not functionally lost but expressed in very specific conditions, that is to say under carbon-depleted and highly aerobic conditions in stationary-phase cells. *Pseudomonas aeruginosa* exhibits a HCO expression pattern similar to *S. oneidensis*, with the promoter of the A-type oxidase being induced under nutrient-starved conditions^18^,^19^. The two transcriptional regulators involved in *cox* gene regulation in *P. aeruginosa*, RpoS and RoxSR, are also present in the genome of *S. oneidensis*; putative binding sites for these two regulators have been identified in the *cox* promoter region, suggesting that the regulatory mechanism involved in the expression of *cox* may be similar in the two bacteria. In the PrrBA system, a RoxSR analog system operating in *Rhodobacter sphaeroides*, PrrA serves as a response regulator, and PrrB^26^ is a membrane sensor which phosphorylates PrrA upon O_2_ deprivation^27^. After growth of *P. aeruginosa* on rich media in highly aerobic conditions, the O_2_ tension decreases in the stationary phase due to the high cell density, which causes RoxSR to repress the *cox* promoter. Conversely, under highly aerobic and nutrient-depleted conditions, biomass remains low when the cells enter the stationary phase and the A-type oxidase is expressed since the high O_2_ concentration prevents the repressor effect of RoxSR^18^,^19^. Another transcriptional regulator that activates the *cox* promoter in *P*. *aeruginosa* is the stationary phase sigma factor RpoS. This factor was found to be involved in survival during carbon starvation in several bacterial species^28^,^29^,^30^,^31^.

In its natural biotope, *S. oneidensis* likely encounters conditions of carbon starvation and highly O_2_ tension. Indeed, *S. oneidensis* was isolated from the sediment of Oneida Lake^32^, a shallow freshwater lake well-mixed by winds where bottom dissolved O_2_ levels are often comparable to the highly aerobic condition defined in this study^33^. In addition, seasonal and spatial variations in nutrient availability can lead to oligotrophic conditions in aquatic systems, especially since Oneida Lake responds quickly to changes in external nutrient influxes due to its short residence time^33^. In these particular environmental conditions, the question arises what would be the physiological advantage(s) bestowed upon *S. oneidensis* by the expression of Cox. Firstly, Cox is likely more energy-efficient than the *cbb*_3_-type oxidase in carbon-starved environments due to a higher proton-pumping stoichiometry, which requires a relative high substrate (O_2_) concentration for optimal operation. Secondly, Cox may play an important role in multicellular structures such as cell aggregates, which resemble biofilms morphologically and physiologically. It was previously reported that *S. oneidensis* cells experiencing highly aerobic carbon starved conditions form aggregates upon addition of calcium chloride at a concentration similar to that found in natural habitats^34^. In *S. oneidensis*, O_2_-dependent autoaggregation was proposed to be a general protective response aimed to reduce the oxidative stress associated with ROS production during aerobic respiration. When cells experience highly aerobic conditions, aggregation may facilitate both the establishment of anaerobic conditions within the structure and the migration into anaerobic zones by gravitation^34^. This would be a relevant strategy to thrive in redox-stratified environments that are liable to promote cellular ROS formation, since *S. oneidensis* is hypersensitive to oxidative stress relative to *E. coli*^35^. We have also observed this multicellular phenotype upon addition of calcium chloride, which occurred only with stationary-phase cells grown under highly aerobic conditions and carbon starvation (data not shown), shown here to specifically induce Cox. We thus hypothesize that *cox* expression may be a component of the defense mechanism against oxidative stress under highly aerobic conditions and carbon starvation in cell aggregates. By consuming O_2_ in the outer part of the aggregate, Cox could contribute to maintaining lower O_2_ levels inside the structure and thus prevent excessive ROS production. Furthermore, Cox could also supply a part of the energy required to maintain the aggregated structure, allowing *S. oneidensis* to better adapt to high O_2_ tensions when carbon sources become scarce, reminding that Cox can extract more energy per unit of carbon by virtue of its high H^+^/e^−^ ratio. Intriguingly, a previously reported global transcriptome analysis comparing aggregated to unaggregated cells revealed a five to eightfold upregulation of the *cox* gene cluster, but this result was not further discussed^34^. Another interesting result revealed by this study^34^ is that the *rpoS* gene is upregulated by a factor of four in these conditions. This strengthens our hypothesis on the putative role of this sigma factor in the regulation of the A-type cytochrome *c* oxidase expression. Unfortunately, no data is available concerning the two-regulatory component PrrBA.

The expression profile of cytochrome *c* oxidases in *S. oneidensis* challenges the generally accepted pattern that in a microorganism that grows aerobically and carries both the A-type and the the C-type oxidase, the former is the main terminal oxidase whereas the latter is only expressed under low O_2_ tensions. It is of note that in the purple photosynthetic bacterium *Rubrivivax gelatinosus*, whereas the *cbb*_3_ oxidase and the *bd*-type quinol oxidase are required for aerobic respiration and for initiation of photosynthesis, the *caa*_3_ oxidase is not induced^36^. As we already noticed, a similar pattern to *S. oneidensis* was reported in *P. aeruginosa*, where the A-type oxidase was not only induced under carbon depletion but also under iron- and nitrogen starvation^18^. In *S. oneidensis* we did not observe aggregates in iron-depleted conditions but only under carbon limitation (data not shown). This supports the hypothesis that the specific expression of Cox in carbon starved cells serves to limit oxidative stress in multicellular structures. Furthermore, carbon starvation appears to be crucial for the expression of genes involved in forming the matrix of *S. oneidensis* biofilms (which is a form of aggregation), and no induction of these genes was observed under nitrogen starvation, in rich or in minimal medium^37^. It should be noted that although the formation of aggregates necessitated the addition of calcium chloride to the medium, since this was at concentrations encountered in the native environments of *S. oneidensis*, this can be considered a limitation of the medium rather than an artificially imposed condition.

Taken together our results indicate that *S. oneidensis* possesses elaborate regulatory mechanisms to fine tune the expression of the different terminal oxidases, enabling it to thrive in stratified environments where O_2_ tension and nutrient concentration often fluctuate. This is further illustrated by the fact that the induction of the *cox* genes is compensated towards lower O_2_ tensions when the *cbb*_3_-type oxidase is lacking, since Cox is detected in all aerobic conditions in the *cbb*_3_-deletion mutant SLL01 under carbon starvation. Further study of these regulatory mechanisms will help to identify and understand the different factors that influence the expression of the terminal oxidases in *S. oneidensis*.

## Methods

### Bacterial strains and growth conditions

Mutant strain SLL01 was constructed *via* in-frame deletion of the gene encoding the catalytic subunit of the *cbb*_*3*_-type cytochrome *c* oxidase (*ccoN*, SO_2364) in *S. oneidensis* MR-1, as previously reported^14^. The strains were maintained on Luria-Bertani (LB) agar plates and were grown in LB medium or in MM3 minimal medium (pH 6.5) containing 30 mM HEPES buffer, 1.5 mM KH_2_PO_4_, 1.5 mM K_2_HPO_4_, 85.5 mM NaCl, 18.7 mM NH_4_Cl, 0.225 mM CaCl_2_, 0.65 mM MgCl_2_, 10 mM NaHCO_3_, 0.5 g.L^−1^ casamino acids, 0.1 g.L^−1^ yeast extract, 20 mM sodium DL-lactate and 1 mL.L^−1^ of trace element solution^38^. The nutrient starvation experiments were carried out in MM3C^−^ (carbon starvation) or in MM3I^−^ (iron starvation), which correspond to MM3 medium without sodium DL-lactate or without FeCl_2_ in the trace element solution, respectively. Cultures were performed at 30 °C in a 30-L BIOSTAT^®^ Cplus-C30-3 fermentor (Sartorius BBI Systems, Germany) as reported previously^14^. The O_2_ partial pressure sensor (InPro^®^ 6820, Mettler-Toledo) was calibrated at 100% in air-saturated medium at 30 °C, which corresponded to an estimated O_2_ concentration of 240 μM. The pO_2_ was regulated at a minimum of 70% (~165 μM O_2_), at 40% (~95 μM O_2_) or at a maximum of 6.5% (~15 μM O_2_) for highly aerobic, aerobic and microaerobic conditions, respectively. During growth, samples were taken from the cultures at the exponential and stationary phases, cells were harvested 10 min at 11400 g and pellets were frozen at −80 °C. For growth parameter measurements, strains were inoculated at an OD_600_ of ~0.1 in 500 mL-flask with baffled base containing 100 mL of medium, and were aerobically grown at 30 °C in an orbital shaker at 200 rpm.

### Preparation and solubilisation of membranes

Membranes were prepared from cell pellets and solubilised with 1% *n*-dodecyl β-D-maltoside (DDM) (w/v) as described before^14^. Protein concentration was determined with the bicinchoninic acid protein assay kit (Sigma-Aldrich).

### Spectral analysis on solubilised membranes

Reduced minus oxidized difference absorbance spectra of DDM-solubilised membranes, pre-incubated with 50 μM potassium cyanide for 10 min, were recorded on a Lambda 25 UV/VIS spectrophotometer (PerkinElmer) at room temperature. Potassium ferricyanide at 1 mM was used as the oxidizing agent and membranes were successively reduced by addition of a few grains of sodium ascorbate and sodium dithionite.

### Heme extraction and HPLC analysis

Non-covalently bound hemes were extracted from non-solubilised membranes as previously described^39^. After evaporating solvent using a vacuum concentrator, the dry materials were stored at −80 °C. Residues were dissolved in 100 μL of acetonitrile +0.05% trifluoroacetic acid (TFA) and passed through a 0.45 μm filter before HPLC analysis. Hemes were separated by reversed-phase HPLC using a Zorbax Eclipse Plus C18 column (5 μm, 150 × 4.6 mm, Agilent Technologies) and detected at 405 nm using a photodiode array detector (Agilent Technologies). The chromatographic separations were performed with a nonlinear gradient of acetonitrile and water with 0.05% TFA at a flow rate of 1 mL.min^−1^. An initial linear gradient of acetonitrile from 45–72.5% over 11 min was followed by a slow increase to 100% acetonitrile (by t = 30 min) and a rapid drop to 45% acetonitrile (by t = 40 min). Hemes were identified by comparison with the retention times of chloroprotoporphyrin IX iron (III) (hemin, which corresponds to heme *b*) and heme *a* extracted from the cytochrome *c* oxidase from bovine heart (both from Sigma-Aldrich).

### Cytochrome c oxidase activity and O_2_ uptake measurements

Reduction of horse heart cytochrome *c* with sodium ascorbate and cytochrome *c* oxidase activity measurements were performed as previously described^14^. Oxygen reductase activity in solubilised membranes was polarographically measured with a Clark-type O_2_ electrode (Oxygraph, Hansatech Instruments) in a stirred volume of 1 mL of 20 mM sodium phosphate buffer pH 7.4, using either ubiquinol-1 or TMPD as electron donor. Ubiquinol-1 was prepared from ubiquinone-1 (Sigma-Aldrich) by resuspension in absolute ethanol and reduction with zinc powder in 5 M hydrochloric acid solution, and stored protected from light at −20 °C. Quinol oxidase activity was determined with 375 μM ubiquinol-1 and 10 mM dithiothreitol. TMPD oxidase activity assays were carried out with 100 μM TMPD and 10 mM sodium ascorbate. Reactions were initiated by the addition of DDM-solubilised membranes and the consumption of O_2_ was recorded at 30 °C. Data were analyzed using the O_2_View software (version 1.02; Hansatech Instruments).

### Separation by Blue-native (BN) gel electrophoresis and in-gel enzymatic activities

BN-gels were performed according to the method of Schägger^40^ as described by Le Laz *et al.* (2014)^14^. Cytochrome *c* oxidase and TMPD oxidase activities were revealed on BN-gels as reported previously^41^,^14^. Protein bands were cut out from the gel and stored at −20 °C before mass spectrometry analysis.

### Protein identification by in-gel digestion and mass spectrometry

Tryptic digestion experiments, electrospray quadrupole time of flight (ESI-Q-ToF) analyses, processing of the spectra and protein search were performed as previously described^14^.

### *Cox* promoter sequence analysis

BDGP Neural Network Promoter Predictor program was used to predict the putative transcription start site (http://www.fruitfly.org/seq_tools/promoter.html) of the *cox* operon. Virtual Footprint software (version 3.0) was used to identify a −10 box for a sigma 70 factor^42^.

## Additional Information

**How to cite this article**: Le Laz, S. *et al.* Expression of terminal oxidases under nutrient-starved conditions in *Shewanella oneidensis*: detection of the A-type cytochrome *c* oxidase. *Sci. Rep.* **6**, 19726; doi: 10.1038/srep19726 (2016).

## Supplementary Material

Supplementary Information

## Figures and Tables

**Table 1:** Growth parameters of *S. oneidensis* cultivated in different media under aerobic conditions.

Medium	Doubling time^a^	Relative yield^b^
MM3	114 +/− 6	1.00
MM3C^−^	237 +/− 12	0.34
MM3I^−^	137 +/− 7	0.50
LB	54 +/− 1	11.36

^a^Expressed in min (mean +/− standard deviation).

^b^Relative yield is defined as the highest optical density at 600 nm in early stationary phase in the different cultures relative to that of the wild-type strain culture in MM3 medium. The standard deviation is less than 3%.

Data were obtained from at least three separated experiments.

**Table 2:** TMPD oxidase activity in solubilised membranes of *S. oneidensis* MR-1 and the *cbb*_3_ oxidase deletion strain (SLL01) under different O_2_ and nutrient conditions.

Strain	Medium	Growth phase^a^	Aerobic conditions	Highly aerobic conditions
*S. oneidensis* *Wild type*	LB	E	90 +/− 6	88 +/− 6
		S	95 +/− 6	88 +/− 3
	MM3	E	52 +/− 1	42 +/− 1
		S	56 +/− 3	33 +/− 1
	MM3I^−^	E	148 +/− 14	132 +/− 8
		S	55 +/− 1	76 +/− 5
	MM3C^−^	E	77 +/− 5	82 +/− 2
		S	158 +/− 12	106 +/− 9
*S. oneidensis* SLL01	LB	E	0	0
		S	0	0
	MM3	E	0	0
		S	0	0
	MM3I^−^	E	0	0
		S	0	0
	MM3C^−^	E	0	0
		S	21 +/− 1	21 +/− 1

^a^Exponential (E) or stationary (S) phase of growth.

Activities are expressed in nmol O_2_.min^−1^.mg protein^−1^. Listed values are averages of at least three separate experiments (mean +/− standard deviation).

**Table 3:** Identification of the cytochrome *c* oxidases by ESI-Q-ToF mass spectrometry in solubilised membranes of *S. oneidensis* MR-1 and the *cbb*_3_ oxidase deletion strain (SLL01) grown under carbon-depleted conditions (MM3C^−^).

O_2_ condition	Strain	Growth phase^a^	Band^b^	Protein name^c^	NCBI enter	Gene	Pept^d^	Cov^e^
Aerobic conditions	*S. oneidensis Wild type*	E	I	No cytochrome *c* oxidase detected				
			II	*cbb*_3_ cyt *c* oxidase sub. II	24348335	*ccoO*	23	47
				*cbb*_3_ cyt *c* oxidase sub. III	24373908	*ccoP*	11	40
		S	I	No cytochrome *c* oxidase detected				
			II	*cbb*_3_ cyt *c* oxidase sub. II	24348335	*ccoO*	17	39
				*cbb*_3_ cyt *c* oxidase sub. III	24373908	*ccoP*	27	65
	*S. oneidensis* SLL01	E	III	No cytochrome *c* oxidase detected				
		S	III	No cytochrome *c* oxidase detected				
			IV	A-type cyt *c* oxidase sub. II	24376079	*coxB*	17	38
			V	A-type cyt *c* oxidase sub. II	24376079	*coxB*	8	22
Highly aerobic conditions	*S. oneidensis Wild type*	E	VI	No cytochrome *c* oxidase detected				
			VIII	*cbb*_3_ cyt *c* oxidase sub. II	24348335	*ccoO*	26	53
				*cbb*_3_ cyt *c* oxidase sub. III	24373908	*ccoP*	13	58
		S	VI	No cytochrome *c* oxidase detected				
			VII	*cbb*_3_ cyt *c* oxidase sub. II	24348335	*ccoO*	17	35
				A-type cyt *c* oxidase sub. II	24376079	*coxB*	11	25
			VIII	*cbb*_3_ cyt *c* oxidase sub. II	24348335	*ccoO*	25	54
				A-type cyt *c* oxidase sub. II	24376079	*coxB*	8	15
	*S. oneidensis* SLL01	S	IX	A-type cyt *c* oxidase sub. II	24376079	*coxB*	12	22
			X	No cytochrome *c* oxidase detected				

^a^Exponential (E) or stationary (S) phase of growth.

^b^Roman numerals refer to the protein bands from the BN gel shown in Fig. 3.

^c^Protein name in NCBI database. Cyt: cytochrome. Sub.: subunit.

^d^Number of peptides detected.

^e^Protein sequence coverage by the matching peptides (%).

These results are representative of three similar experiments.

**Table 4:** Quinol oxidase activity in solubilised membranes of *S. oneidensis* MR-1 under different O_2_ and nutrient conditions.

Medium	Growth phase^a^	Aerobic conditions	Highly aerobic conditions
LB	E	9 +/− 1	77 +/− 7
	S	20 +/− 1	79 +/− 5
MM3	E	396 +/− 35	201 +/− 10
	S	319 +/− 9	240 +/− 10
MM3I^−^	E	235 +/− 23	0
	S	137 +/− 8	0
MM3C^−^	E	521 +/− 13	0
	S	0	0

^a^Exponential (E) or stationary (S) phase of growth.

Activities are expressed in nmol O_2_.min^−1^.mg protein^−1^. Listed values are averages of at least three separate experiments (mean +/− standard deviation).
