# Supplementary material for: Expression of terminal oxidases under nutrient-starved conditions in Shewanella oneidensis: detection of the A-type cytochrome c oxidase
Source: Sci Rep. 2016 Jan 27;6:19726. doi: 10.1038/srep19726 (PMC4728554; doi:10.1038/srep19726)
Supplement: Supplementary Information [file srep19726-s1.pdf]

Supplementary information file

Expression of terminal oxidases under nutrient-starved conditions in *Shewanella oneidensis*: detection of the A-type cytochrome *c* oxidase

Sébastien LE LAZ[1], Arlette KPEBE[1], Marielle BAUZAN[2], Sabrina LIGNON[3], Marc ROUSSET[1] and Myriam BRUGNA[1*]

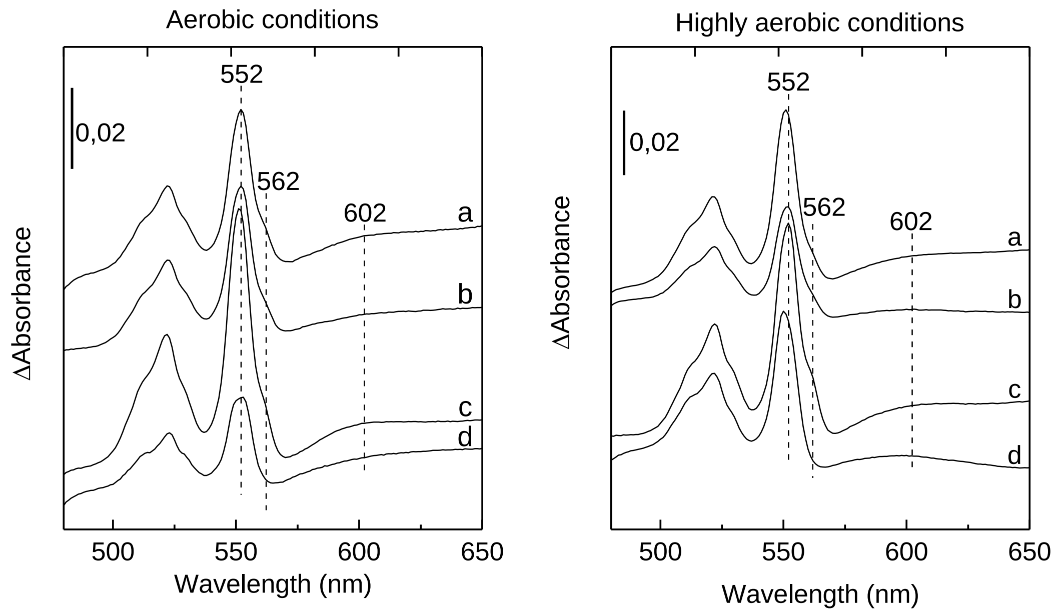

Figure S1: Reduced minus oxidized difference absorbance spectra of solubilised membranes from *S. oneidensis* wild-type and the *cbb*$_3$ oxidase deletion strain (SLL01) grown under different O_2 and nutrient conditions. The spectra were recorded at room temperature in the presence of 50 µM KCN. The membranes were oxidized with potassium ferricyanide and reduced with sodium ascorbate. The concentration of proteins was 6.0 mg.mL^{-1}. The vertical bar indicates the absorption scale. a: wild-type in LB at exponential phase. b: wild-type in minimal medium (MM3) at exponential phase. c: wild-type in iron-depleted medium (MM3I^{-}) at exponential phase. d: SLL01 strain in carbon-depleted medium (MM3C^{-}) at exponential phase.

a

Strain	TMPD oxidase activity	Quinol oxidase activity
**MR-1**	227 +/- 16	0
**SLL01**	17 +/- 1	0

b

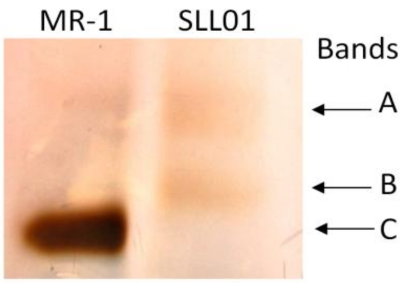

c

Strain	Band[a]	Protein name[b]	NCBI enter	Gene	Pept[c]	Cov[d]
**MR-1**	A	*cbb*$_3$ cyt. *c* oxidase sub. II	24348335	*ccoO*	15	57
		cbb$_3$ cyt. *c* oxidase sub. III	24373908	*ccoP*	18	62
		bd quinol oxidase sub. I	24349471	*cydA*	28	44
	C	*cbb*$_3$ cyt. *c* oxidase sub. I	24373911	*ccoN*	14	18
		cbb$_3$ cyt. *c* oxidase sub. II	24348335	*ccoO*	21	50
		cbb$_3$ cyt. *c* oxidase sub. III	24373908	*ccoP*	12	39
		bd quinol oxidase sub. I	24349471	*cydA*	12	13
**SLL01**	A	*bd* quinol oxidase sub. I	24349471	*cydA*	32	22
	B	A-type cyt *c* oxidase sub. II	24376079	*coxB*	17	30
		bd quinol oxidase sub. I	24349471	*cydA*	24	21

Figure S2: TMPD and quinol oxidase activities, and identification of the terminal oxidases by mass spectrometry in solubilised membranes of *S. oneidensis* MR-1 and the *cbb*$_3$ oxidase deletion strain (SLL01) grown in carbon-depleted medium (MM3C$^-$) under microaerobic conditions, in stationary growth phase. a. TMPD and quinol oxidase activities expressed in nmol O_2.min^{-1}.mg protein^{-1}. Listed values are averages of at least three separate experiments (mean +/- standard deviation). b. In-gel detection of cytochrome *c* oxidase activity in BN-gel. Total proteins (130 μg) were loaded on a 5-15% polyacrylamide gel. Bands of activity are indicated by arrows. c. Identification of the terminal oxidases by ESI-Q-ToF mass spectrometry. [a] Band letters refer to the protein bands from the BN gel shown in panel b. [b] Protein name in NCBI database. Cyt. : cytochrome. Sub. : subunit. [c] Number of peptides detected. [d] Protein sequence coverage by the matching peptides (%).

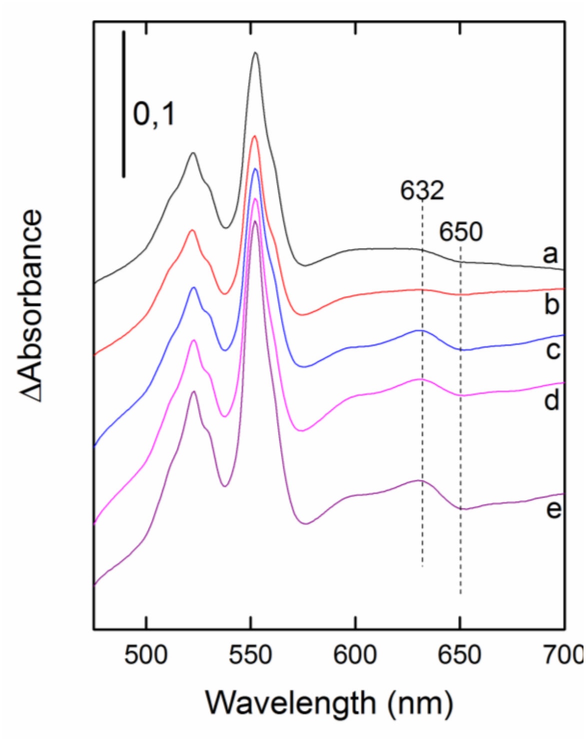

Figure S3: Reduced minus oxidized difference absorbance spectra of solubilised membranes from *S. oneidensis* wild-type grown under different O_2 and nutrient conditions. The spectra were recorded at room temperature in the presence of 50 µM KCN. The membranes were oxidized with potassium ferricyanide and reduced with sodium dithionite. The concentration of proteins was 6.0 $mg.mL^{-1}$. The peak at 632 nm and the trough at 650 nm arise from the heme *d* of the *bd*-quinol oxidase [15]. The vertical bar indicates the absorption scale. a: in $MM3I^-$ at exponential phase and highly aerobic condition. b: in $MM3I^-$ at stationary phase and highly aerobic condition. c: in $MM3C^-$ at exponential phase and highly aerobic condition. d: in $MM3C^-$ at stationary phase and highly aerobic condition. e. in $MM3C^-$ at exponential phase and aerobic condition.

Table S1: Identification of the cytochrome *c* oxidases by ESI-Q-ToF mass spectrometry in solubilised membranes of the wild type *S. oneidensis* MR-1, grown in rich (LB), minimal (MM3) and iron-depleted (MM3I$^-$) media under aerobic and highly aerobic conditions.

O_2 tension	Medium	Growth phase[a]	Band[b]	Protein name[c]	NCBI enter	Gene	Pept[d]	Cov[e]
**Aerobic conditions**	**MM3**	E	I	No cytochrome *c* oxidase detected				
			II	cbb_3 cyt. *c* oxidase sub. II	24348335	*ccoO*	11	37
				cbb_3 cyt. *c* oxidase sub. III	24373908	*ccoP*	14	56
		S	I	No cytochrome *c* oxidase detected				
			II	cbb_3 cyt. *c* oxidase sub. II	24348335	*ccoO*	7	33
				cbb_3 cyt. *c* oxidase sub. III	24373908	*ccoP*	14	48
	**MM3I$^-$**	E	I	No cytochrome *c* oxidase detected				
			II	cbb_3 cyt. *c* oxidase sub. II	24348335	*ccoO*	12	38
		S	I	No cytochrome *c* oxidase detected				
			II	cbb_3 cyt. *c* oxidase sub. II	24348335	*ccoO*	11	39
				cbb_3 cyt. *c* oxidase sub. III	24373908	*ccoP*	9	43
**Highly aerobic conditions**	**LB**	E	VI	No cytochrome *c* oxidase detected				
			VIII	cbb_3 cyt. *c* oxidase sub. II	24348335	*ccoO*	17	41
				cbb_3 cyt. *c* oxidase sub. III	24373908	*ccoP*	16	61
		S	VI	No cytochrome *c* oxidase detected				
			VIII	cbb_3 cyt. *c* oxidase sub. II	24348335	*ccoO*	10	31
				cbb_3 cyt. *c* oxidase sub. III	24373908	*ccoP*	14	56
	**MM3**	E	VI	No cytochrome *c* oxidase detected				
			VIII	cbb_3 cyt. *c* oxidase sub. II	24348335	*ccoO*	15	58
				cbb_3 cyt. *c* oxidase sub. III	24373908	*ccoP*	22	47
		S	VI	No cytochrome *c* oxidase detected				
			VIII	cbb_3 cyt. *c* oxidase sub. II	24348335	*ccoO*	13	51
				cbb_3 cyt. *c* oxidase sub. III	24373908	*ccoP*	13	40
	**MM3I$^-$**	E	VI	No cytochrome *c* oxidase detected				
			VIII	cbb_3 cyt. *c* oxidase sub. II	24348335	*ccoO*	10	26
				cbb_3 cyt. *c* oxidase sub. III	24373908	*ccoP*	12	51
		S	VI	No cytochrome *c* oxidase detected				
			VIII	cbb_3 cyt. *c* oxidase sub. II	24348335	*ccoO*	17	43
				cbb_3 cyt. *c* oxidase sub. III	24373908	*ccoP*	10	43

[a] Exponential (E) or stationary (S) phase of growth.
[b] Roman numerals refer to the protein bands from the BN gel shown in Fig.3.
[c] Protein name in NCBI database. Cyt. : cytochrome. Sub. : subunit.
[d] Number of peptides detected.
[e] Protein sequence coverage by the matching peptides (%).

Table S2: Identification by ESI-Q-ToF mass spectrometry of the subunit I of the *bd*-quinol oxidase (CydA, NCBI enter: 24349471) in solubilised membranes of *S. oneidensis* MR-1 grown in MM3C$^-$ or MM3I$^-$ under aerobic or highly aerobic conditions.

O_2 tension	Growth medium	Growth phase[a]	Peptides[b]	Coverage[c]
**Aerobic conditions**	MM3C$^-$	S	52	30
**Highly aerobic conditions**	MM3C$^-$	E	51	28
		S	38	29
	MM3I$^-$	E	16	20
		S	21	22

[a] Exponential (E) or stationary (S) phase of growth.
[b] Number of peptides detected.
[c] Protein sequence coverage by the matching peptides (%).
Cyd A was identified from bands cut out from the BN-gels shown in Fig. 3.
